# Utilization of Banana Fiber-Reinforced Hybrid Composites in the Sports Industry

**DOI:** 10.3390/ma13143167

**Published:** 2020-07-16

**Authors:** Ans Al Rashid, Muhammad Yasir Khalid, Ramsha Imran, Umair Ali, Muammer Koc

**Affiliations:** 1 Division of Sustainable Development (DSD), College of Science & Engineering (CSE), Hammad Bin Khalifa University (HBKU), Qatar Foundation (QF), Education City, Doha, Qatar; mkoc@hbku.edu.qa; 2 Department of Mechanical Engineering, University of Management & Technology, Lahore 54782, Pakistan; yasir.khalid@skt.umt.edu.pk (M.Y.K.); ramshaimran880@gmail.com (R.I.); cadetumair786@gmail.com (U.A.); 3 Department of Mechanical Engineering, Institute of Space Technology, Islamabad 44000, Pakistan; 4 Department of Engineering Science, University of Greenwich, London SE10 9LS, UK

**Keywords:** hockey, banana fiber-reinforced composites, hybrid composites, finite element analysis

## Abstract

The sports industry is an ever-growing sector worldwide. With technological advancements in information technologies, the sports industry has merged with the entertainment industry, reaching and influencing billions of people globally. However, to ensure and advance the safety, security, and sustainability of the sports industry, technological innovations are always needed in several manufacturing and materials processes to achieve cost-effectiveness, efficiency, durability, reusability, and recyclability of products used in this industry. For example, 90% of the field hockey equipment produced in the world comes from Sialkot, Pakistan. Most export quality field hockey equipment is currently produced via reinforcement of glass/carbon fibers in epoxy resin. The current study aimed to introduce new materials for field hockey equipment to reduce manufacturing costs and the environmental impact of synthetic materials, without comprising the quality of the final product. Our literature review on natural fibers revealed that they offer excellent and compatible mechanical properties. Based on extensive experimental studies, we concluded that banana fiber reinforced hybrid composites could be an alternative to pure glass fiber reinforced composites, with comparable and even higher load withstanding capabilities. Using banana fiber reinforced hybrid composites for the fabrication of hockey products would cut costs and lower the environmental impact stemming from the uses of biodegradable organic materials. It will also lead to the development of a domestic economy based on domestic resources.

## 1. Introduction

The sports industry is an ever-growing sector all around the world. With technological advancements in information technologies, the sports industry has merged with and become a significant entertainment industry reaching and influencing billions of people globally. However, in order to ensure and advance the safety, security, and sustainability of the sports industry, technological innovations are always needed in the manufacturing and materials processes to attain cost-effectiveness, efficiency, durability, reusability, and recyclability of the products used in this industry. Although there are players like China and India in the fabrication and export of field hockey products around 90% of hockey equipment in the world is produced in Sialkot, Pakistan [1]. Businesses are investing in the research and development of processes and materials to compete in the international market with economic and quality products. Most export quality, field hockey products are made by reinforcement of glass/carbon fibers in epoxy resin. Composites usually have two different phases at the microscopic scale and it is essential to determine these integrants. Constituents with inferior physical and mechanical properties (the matrix) are combined with constituents having superior mechanical properties (reinforcement). Fiber-reinforced plastics possess some superior mechanical properties, including density, stiffness, and strength. These would unquestionably be the design drivers for the selection of materials in transportation [2], sports, medical, construction, and packaging industries, as well as in consumer applications [3].

Increased demand for materials with a high strength to weight ratio has led researchers to develop novel materials. Although synthetic fiber-reinforced polymer composites have significant mechanical strength, they also have some genuine drawbacks like comparatively high costs, high density, poor recycling, and non-biodegradable properties. Consequently, there have been significant developments in recent years in the use of natural fiber-reinforced composites. Natural fibers are being considered as a tenable substitute to synthetic fibers as they offer many advantages, including low cost, low density, biodegradability, reusability, and recyclability [4,5,6,7,8]. Banana, coir, flax, hemp, jute, kenaf, and pineapple leaf are the most common example of natural fibers.

Natural fibers reinforced polymer composites (NFRPC) do not offer superior mechanical properties than synthetic fibers reinforced polymer composites (FRPC). Venkateshwaran et al. [9] found that to enhance the properties of natural fibers; they can be mixed with conventional fibers to produce hybrid composites.

Weed et al. [10] found that banana fiber-reinforced composites had comparatively weak bonding between fiber and matrix. Jacob et al. [11] observed that banana fibers had a hydrophilic nature, and there was weak bonding between the matrix and fiber when the banana fiber was combined with the hydrophobic matrix. Paul et al. [12] described methods that could be utilized for the improvement of bonding. The first method was to make the fiber surface rough so that the polymer can anchor at the mechanical anchoring sites. The second procedure was a chemical treatment that could be used to modify the fiber surface for the reduction in its hydrophilicity, to improve its bonding with the matrix.

Li et al. [13] found chemical treatments that could be used to improve the surface of the fibers. Silane treatment and alkali treatment are the most commonly known treatments. Peroxide treatment and permanganate treatment are also among popular chemical treatments, while maleic anhydride can be used as a coupling agent. Alkali treatment of natural fibers is also known as mercerization. Summerscales et al. [14] observed that this process could reduce fiber diameter, and the reduction in the diameter increased the aspect ratio. Consequently, better bonding between the matrix and the fiber was achieved, and the material was improved mechanically. Singh et al. [15] found that the tensile strength of composites increased by 50% after alkali treatment.

Hybrid composites are comparatively more flexible. Within a few years, the use of the natural fiber reinforcement in plastic composites has increased [16,17,18,19]. Venkateshwaran et al. [9] found that only glass fiber and carbon fiber improved their mechanical properties from hybridization. Consequently, glass fiber or carbon fiber hybrids with banana or sisal fiber offer low-cost applications. It was found that natural fibers reduced the weight of composites by 10%, and the energy used for the production was reduced by 80%, while there was a 5% reduction in the cost of components.

Dhawan et al. [20] made the epoxy and polyester-based glass fiber reinforced composites and studied natural filler effects. They found that better results were obtained from natural fillers in polymer-based composites. The authors also found that coir in composites gave comparatively superior mechanical properties. Fombuena et al. [21] found that calcium carbonate from seashells can be added to enhance the mechanical properties of materials.

Mishra et al. [22], Joseph et al. [23], Pothan et al. [24], Uday Kiran et al. [25], and Haneefa et al. [26] observed the combination of banana fibers in thermoset plastics. Haneefa et al. [26] analyzed the combination of banana fiber and glass fiber and found that the tensile strength and Young’s modulus of the material were improved.

Prasad et al. [27] used a different volume fraction between the glass fiber and natural fibers (banana and jute) with specimens of diverse volume segments, i.e., 40%, 45%, 50%, and 55%. Using the experimental data, finite element analysis was carried out to compare the tensile strength of the jute and banana fiber reinforced resin-based hybrid.

Sanjay et al. [28] produced a hybrid composite of jute and glass fibers and they found that the mechanical properties of the composites increased. Jeyanthi et al. [29] fabricated a hybrid composite of kenaf and glass fibers with epoxy resin using the hot impregnation method, where this technique also increased the mechanical properties of the material. The authors also found that impact strength was increased by adding some additives. Gopinath et al. [30] concluded that polyester reinforced with jute fibers had inferior mechanical properties compared to jute/epoxy composites.

In previous studies, most of the research was related to individual reinforcement rather than the mixing of natural fiber with synthetic fiber. This study aimed at introducing novel materials for field hockey products to reduce manufacturing costs and the environmental impact of synthetic materials without comprising product quality. The flexural properties of banana and glass hybrid composites were evaluated experimentally and numerically, and the results were compared to the glass fiber reinforced composite which is used conventionally in hockey equipment production.

## 2. Materials and Methods

In this study, authors used plain-woven E-glass fibers purchased from the National Textile University, Faislabad, Pakistan, and unidirectional banana fibers obtained from Organic Fibers, Karachi, Pakistan. The specimens were prepared using the hand layup method. A particular stacking sequence generally used in hockey products production was selected and the specimens were manufactured with a different weight content of banana and glass fibers based on the available literature [31]. Details about materials, manufacturing process, mechanical testing, and numerical modeling are explained in the next section.

### 2.1. Materials (Matrix and Reinforcements) and Manufacturing of the Composite

**Figure 1:**
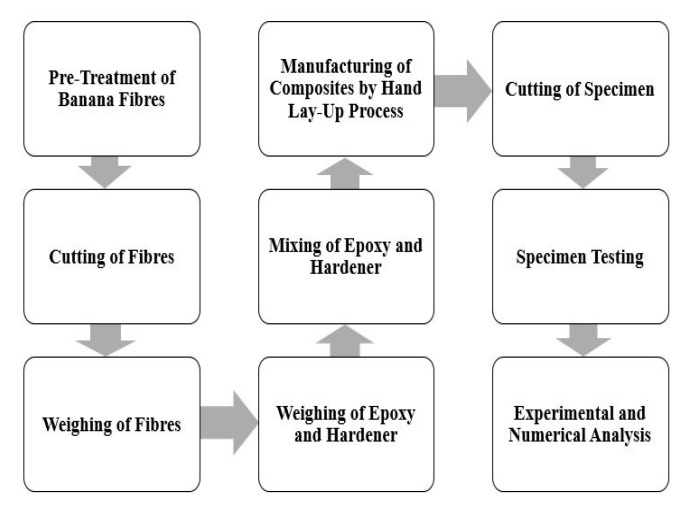
The layout of the process.

**Figure 2:**
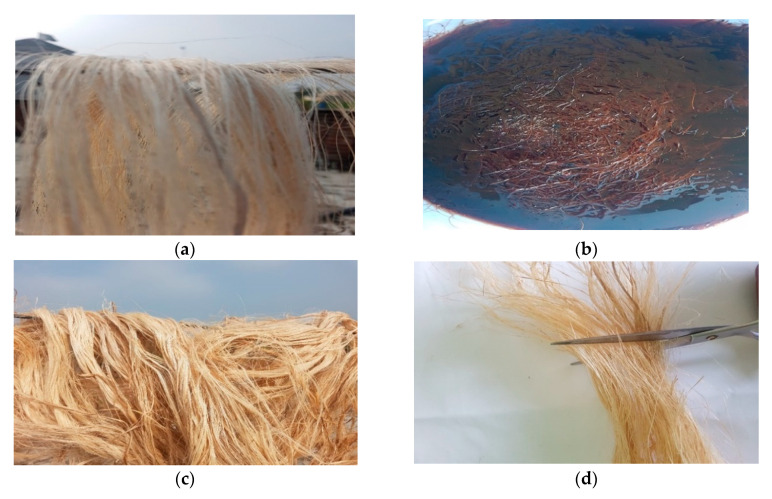
The manufacturing process of the banana/glass fiber hybrid composite. (**a**) Drying before chemical treatment; (**b**) chemical treatment; (**c**) post treatment drying; (**d**) cutting banana fiber.

The mechanical properties of the matrix and reinforcements used for the fabrication of materials are reported in Table 1. For specimen preparation, the banana fibers were first treated with 5% NaOH and 95% distilled water by volume solution for 24 h. Then they were cut into the required dimensions and weighed. Next, acetone was used to wash the glass mold and then it was dried and spattered with a mold releasing spray. Afterward, peel ply was applied to the spattered region. The fibers were placed on the glass mold and epoxy was applied to it with the help of a brush. Subsequently, a roller was used to gain uniform distribution and to remove the excess epoxy from the glass surface. Then, a weight was placed on the glass mold for better curing. Minor variations in laminate thicknesses were observed between different stacking sequences, as the wet layup method cannot guarantee 100% accuracy. The layout of the process is presented in Figure 1. Steps involved in the pre-treatment of the fibers are shown in Figure 2. After the laminate preparation, the samples were taken out of the glass mold and cut into the required dimensions as per the ASTM Standard D7264 [32] and then kept in airtight bags for further experimentation. Details of the stacking sequence, sample numbers, the weight of specimens, and description of the material are presented in Table 2 and sample dimensions are reported in Table 3. Banana fibers are lightweight substitutes for glass fibers. Therefore, adding more layers of banana fibers reduced the weight of the composite material. The reason for the significant difference between B1 and B3 layer composites was due to the hydrophilicity of natural fibers, as they were not able to absorb the same content of epoxy as compared to glass fibers.

### 2.2. Mechanical Testing

**Figure 3:**
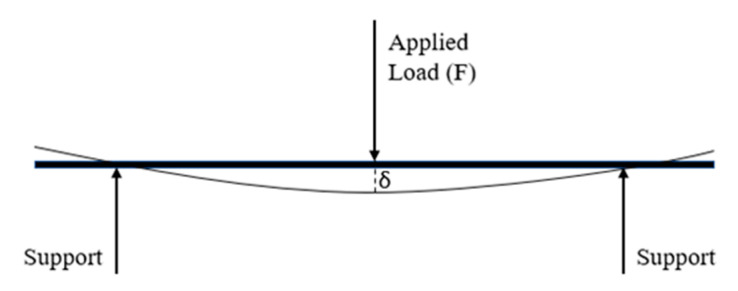
Schematic diagram of 3 Point Bending Test.

According to ASTM Standard D7264, a three-point bending test was conducted to evaluate the flexural properties. The universal testing machine was used to perform the bending test as presented in Figure 3.

Load versus displacement data extracted from the universal testing machine was used to determine the stresses and strains during loading using Equations (1) and (2).

(1) $$\sigma =3PL⁄\left(2bh^{2}\right)$$

where, *σ* = stress (MPa), *P* = force applied (N), *L* = length of supporting span, *b* = beam breadth, *h* = beam thickness.

(2) $$\epsilon =6\delta h⁄L^{2}$$

where, *ϵ* = strain, *δ* = deflection at mid-span, *L* = length supporting span, *h* = beam breadth.

### 2.3. Numerical Simulation

Numerical analysis of the banana and glass fiber reinforced hybrid composites was conducted using ABAQUS (v6.14 by Dassault Systemes, Vélizy-Villacoublay, France) to validate the experimental results. ABAQUS is more compatible with the fiber composites analysis than any other simulation tool. First, the specimens were designed on ABAQUS according to the geometry of the specimen used in experimentation. The material for the supports and rollers was considered as a non-deformable rigid material. In the next step, the total layers were created symmetrically, and the material was assigned by defining properties of the materials. Engineering constants used to define lamina properties are reported in Table 4. In the composite layup module, material orientation, thickness, and the rotation angle of fibers could be defined. The mesh should be fine enough for accurate results; therefore, finer mesh promises more reliable results. The boundary conditions were defined to identify constraints during the simulation. The stress-stain curve from the midpoint of the plate could be extracted at that instance of maximum deformation.

## 3. Results and Discussion

### Flexural Test Results

**Figure 4:**
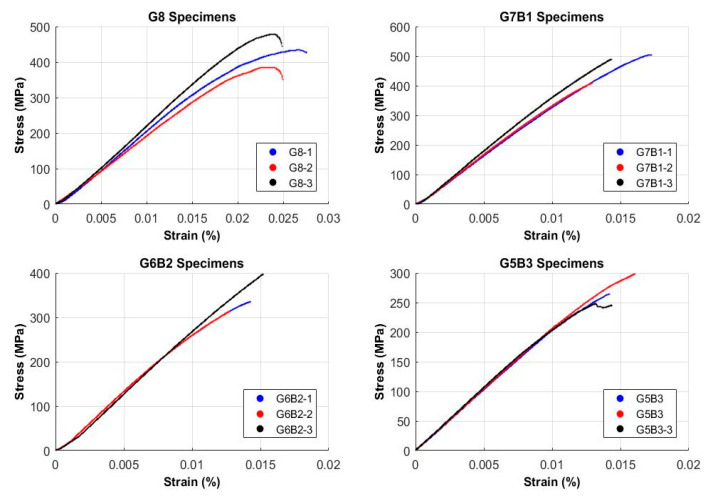
Strain versus stress plots.

Load-displacement data can be extracted from the universal testing machine. Stress-strain values can be calculated using the formulas mentioned above, and diagrams for all specimens tested are shown in Figure 4. Minor deviations in the stress-strain curves were observed for G8 samples but plots for the hybrid composites were consistent. It was possible to calculate the flexural modulus from the stress-strain curves but only the flexural strength was considered in the interest of the study. It is worth mentioning that higher strain values were observed for pure glass fiber composite samples as compared to hybrid composites. The addition of banana fibers caused limitations to the ductility of the material, thereby limiting its applications desiring ductile materials.

**Figure 5:**
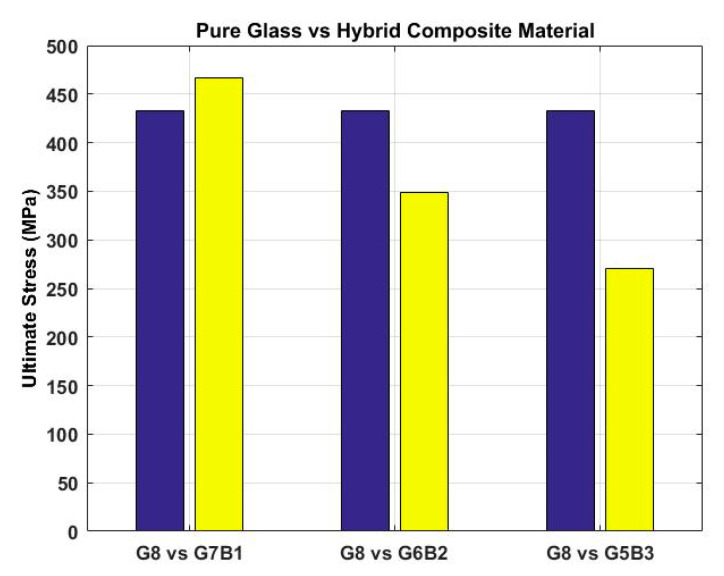
G8 versus hybrid materials.

Ultimate stresses for glass fiber reinforced composite materials were compared with hybrid composite materials (Figure 5). The results from experimentation showed that the hybrid composite G7B1 possessed a higher flexural strength of 470 MPa. Ultimate stresses for different compositions of composite materials were arranged in the descending order below.

G7BI>G8>G6B2>G5B3

The above sequence showed hybridization increased flexural strength up to a certain percentage of banana fiber. Lower strength was observed when more layers of banana fiber were added, due to weak mechanical bonding owing to the hydrophilic nature and lower flexural strength.

**Figure 6:**
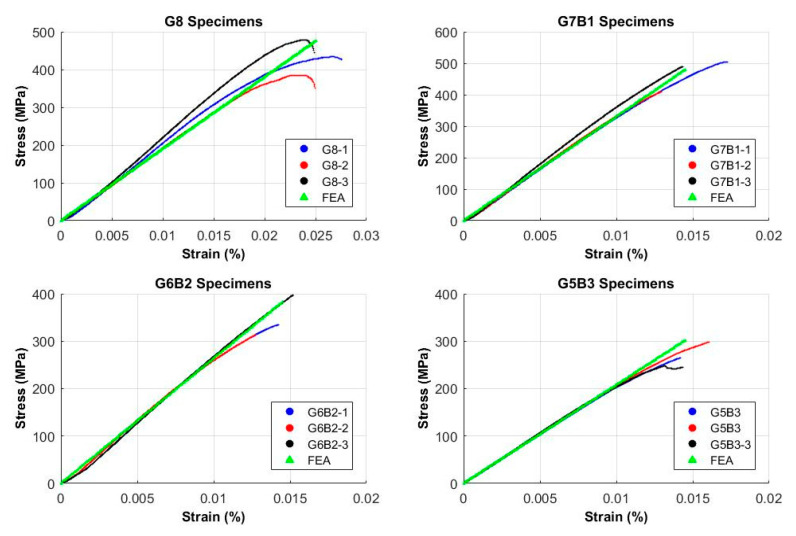
Experimental versus finite element analysis (FEA) results.

A comparison of experimental results and the Finite Element Analysis (FEA) simulations is presented in Figure 6. The numerical simulation predicted experimental results with an error of approximately 10–15% for all stacking sequences. The reason for this error was the presence of voids, non-uniform fiber distribution, and possible delamination of layers in the hand layup technique. FEA tool (ABAQUS) can correctly predict the linear elastic behavior of material for different stacking sequences. Nonetheless, advanced simulations can be performed to predict the plastic behavior of the composites for applications involving fatigue applications.

## 4. Conclusions & Future Recommendations

Based on experimental and numerical observations, we concluded that a hybrid polymer matrix composite with the reinforcement of banana fiber could withstand higher loads and stresses as compared to pure glass fiber reinforced composites. G7B1 composite can withstand higher loads as compared to the G8 composite, while G6B2 composite shows relatively lower strength with respect to the G8 composite. The possible reason for G7B1 results showing higher flexural strength than G8 is a strong bonding between the plies. Increasing the number of banana-fiber layers reduces the strength of the material. As banana fiber has poor properties compared to glass fiber such as uniformity, hydrophilic phenomenon, and strength, it can only be used in hybrid composites at a certain optimized weight percentage, such that adding more layers of banana fibers drastically decreases the mechanical properties. Although there is a limitation to the application of natural fibers, further research should be carried out to enhance their applicability in industrial products.

From the experimental results, we observed that the addition of banana fiber in the polymer matrix resulted in brittleness of the material. It is recommended to add coir fiber along with banana fiber to enhance the ductility of the material where brittleness is not desired [35].

It can be concluded from the numerical simulation that FEA tools can correctly predict the mechanical behavior of the composite materials and can be used to analyze innovative materials further.

Newly designed materials could be a replacement for synthetic fibers conventionally used in field hockey equipment production due to the considerable economic and environmental benefits. Further studies can be performed to analyze such innovative and green materials to improve the mechanical properties of natural fibers to replace synthetic materials with eco-friendly materials completely.

## Figures and Tables

**Table 1:** Mechanical properties of the materials.

Mechanical Properties	EPOTEC YD-134 [33]	Banana Fibers [10]	E-Glass Fibers [34]
Density ($\mathrm{kg}/m^{3}$)	700	1350	2550
Tensile Strength (MPa)	–	392–677	3100–3800
Young’s Modulus (GPa)	3.1	27–32	72.5–75.5
Shear Modulus (GPa)	1.25	12.5	33
Failure Strain (%)	–	1–3	4.70
Poisson’s Ratio	0.24	0.23	0.21

**Table 2:** Details of the specimens.

Stacking Sequence	Samples	Weight (g)	Description
G8	G8-1	21.96	8 layers of E-glass fibers.
	G8-2	21.88	
	G8-3	21.93	
G4B1G3 (G7B1)	G4B1G3-1	21.68	4 layers of E-glass fibers, 1 layer of banana fibers, and then 3 layers of E-glass fibers.
	G4B1G3-2	21.72	
	G4B1G3-3	21.66	
G3B2G3 (G6B2)	G3B2G3-1	19.10	3 layers of E-glass fibers, 2 layers of banana fibers, and then 3 layers of E-glass fibers.
	G3B2G3-2	19.07	
	G3B2G3-3	19.11	
G3B3G2 (G5B3)	G3B3G2-1	17.31	3 layers of E-glass fibers, 3 layers of banana fibers, and then 2 layers of E-glass fibers.
	G3B3G2-2	17.25	
	G3B3G2-3	17.29	

**Table 3:** Sample dimensions after cuttings.

Stacking Sequence	Thickness (mm)	Total Length (mm)	Average Width (mm)
G8	2.82	80	14.84
G7B1	2.85	80	14.71
G6B2	2.93	80	14.55
G5B3	3.08	80	14.65

**Table 4:** Engineering constants used in simulation.

Physical Property	Glass/Epoxy Lamina	Banana/Epoxy Lamina
Density (kg⁄m^3^)	2550	1350
$E_{1}$ (MPa)	13,010	7828
$E_{2}$ (MPa)	13,010	3400
$E_{3}$ (MPa)	4953	3400
$G_{12}$ (MPa)	2850	1250
$G_{23}$ (MPa)	1795	1250
$G_{13}$ (MPa)	1795	1100
$v_{12}$	0.22	0.23
$v_{23}$	0.3	0.23
$v_{13}$	0.3	0.32

## Data Availability

The raw/processed data required to reproduce these findings cannot be shared at this time as the data also forms part of an ongoing study.

## Abbreviations

NFRPC	Natural fiber reinforced polymer composites
FRPC	Fiber-reinforced polymer composites
FEA	Finite Element Analysis
